# Hair cortisol concentration reflects the life cycle and management of grey wolves across four European populations

**DOI:** 10.1038/s41598-022-09711-x

**Published:** 2022-04-05

**Authors:** Patrícia Pereira, Núria Fandos Esteruelas, Mónia Nakamura, Helena Rio-Maior, Miha Krofel, Alessia Di Blasio, Simona Zoppi, Serena Robetto, Luis Llaneza, Emilio García, Álvaro Oleaga, José Vicente López-Bao, Manena Fayos Martinez, Jasmine Stavenow, Erik O. Ågren, Francisco Álvares, Nuno Santos

**Affiliations:** 1 CIBIO/InBIO-Research Center in Biodiversity and Genetic Resources, University of Porto, Vairão, Portugal; 2 Department of Biology, Faculty of Sciences, University of Porto, Porto, Portugal; 3 Department of Forestry, Biotechnical Faculty, University of Ljubljana, Ljubljana, Slovenia; 4 Istituto Zooprofilattico Sperimentale del Piemonte, Liguria e Valle d’Aosta, Turin, Italy; 5 A.S.L. TO3, Azienda Sanitaria Locale di Collegno e Pinerolo, Turin, Italy; 6 CeRMAS, National Reference Centre for Wild Animal Disease, Istituto Zooprofilattico Sperimentale del Piemonte, Liguria e Valle d’Aosta, Quart, Aosta, Italy; 7 A.RE.NA, Asesores en Recursos Naturales, S.L, Lugo, Spain; 8 Research Unit of Biodiversity (UO/CSIC/PA), Oviedo University, Mieres, Spain; 9 SERPA, Sociedad de Servicios del Principado de Asturias S.A., Gijón, Asturias Spain; 10 Centro de Recuperación de Fauna Silvestre de Cantabria, TRAGSATEC, Cantabria, Spain; 11 Department of Pathology and Wildlife Diseases, National Veterinary Institute, Uppsala, Sweden

**Keywords:** Conservation biology, Ecophysiology

## Abstract

The grey wolf (*Canis lupus*) persists in a variety of human-dominated landscapes and is subjected to various legal management regimes throughout Europe. Our aim was to assess the effects of intrinsic and methodological determinants on the hair cortisol concentration (HCC) of wolves from four European populations under different legal management. We determined HCC by an enzyme-linked immune assay in 259 hair samples of 133 wolves from the Iberian, Alpine, Dinaric-Balkan, and Scandinavian populations. The HCC showed significant differences between body regions. Mean HCC in lumbar guard hair was 11.6 ± 9.7 pg/mg (range 1.6–108.8 pg/mg). Wolves from the Dinaric-Balkan and Scandinavian populations showed significantly higher HCC than Iberian wolves, suggesting that harvest policies could reflected in the level of chronic stress. A significant negative relationship with body size was found. The seasonal, sex and age patterns are consistent with other studies, supporting HCC as a biomarker of chronic stress in wolves for a retrospective time frame of several weeks. Our results highlight the need for standardization of sampling and analytical techniques to ensure the value of HCC in informing management at a continental scale.

## Introduction

The mechanisms by which the degradation of natural habitats influences wild animals can require an understanding of their physiological responses to stressors^1^. Stressors can be defined as somatic or psychological challenges to homeostasis that first activate the sympathetic nervous system and then the hypothalamus–pituitary–adrenal (HPA) axis^2^. As a result, stressors increase the levels of glucocorticoids in the organism^3^. While glucocorticoids are commonly used as biomarkers of chronic stress, their usefulness in wildlife is limited due to the influence of short-term stressors, such as capture, on their concentrations in the most commonly used biological matrixes, namely serum, feces, saliva, and urine^4–6^. In this regard, the quantification of cortisol, the major glucocorticoid in many mammal species, in hair is emerging as a useful biomarker of chronic stress in wildlife^4,6^.

While the mechanisms by which cortisol is incorporated in hair remain to be demonstrated, hair cortisol concentration (HCC) is hypothesized to reflect the integrated biologically active free cortisol fraction (not bound to glucocorticoid-binding globulin) rather than the total cortisol concentration in serum^4,7,8^. Indeed, several studies have found good correlations between cortisol concentration in hair and simultaneous serial samples of conventional biological matrices such as blood, saliva, or feces^8–10^.

Hair can be preferred over other biological matrices for ethical reasons. Hair samples can be obtained non-invasively^4,11^, thus addressing the pressing issues of animal welfare in wildlife research^12^. Additionally, hair collection is simple and inexpensive when performed on specimens in archives and on recently dead animals^13,14^. This facilitates achieving larger sample sizes and wider spatial–temporal coverage than with invasive sampling.

Cortisol in blood or feces reflects the activation of the HPA axis from minutes to a few days before sample collection^6,9^. In contrast, cortisol is hypothesized to be integrated into the hair over a longer time frame, providing a picture of the activation of the HPA axis over a time frame of weeks to months^4,6,11^. Hence, this method offers an opportunity to study the physiological responses of wildlife to natural processes and potential long-term stressors such as social interactions, infectious diseases, or human perturbation^15^.

The grey wolf (*Canis lupus*; hereafter, wolf) inhabits different human-dominated landscapes across its range^16,17^. While the historical range of the wolf covered most of the northern hemisphere, by the end of the nineteenth century it was exterminated from most of western, central, and northern Europe^16,18^. In the last decades, the wolf has recolonized large parts of its former European range, including human-dominated landscapes, with an average human density of 36.7 inhabitants/km^2^^16^. Wolf management regimes vary across Europe, with the species being fully protected in parts of the Iberian and Alpine population ranges, while hunted or subjected to legal population control in much of the Scandinavian and Dinaric-Balkan ranges^19,20^. This makes European wolf populations a relevant model for studying conservation physiology.

The aim of this study was to assess the effect of intrinsic determinants (sex, age, body condition, body structural size, month, and cause of death/capture, and wolf population) on HCC, as determined by enzyme-linked immune assay (ELISA), of European wolves from the Iberian, Alpine, Dinaric-Balkan, and Scandinavian populations while controlling for the confounding effect of variables related to the methods employed (weight and length of hair used for cortisol extraction, sample storage time, body region and methanol evaporation protocol).

## Results

**Figure 1:**
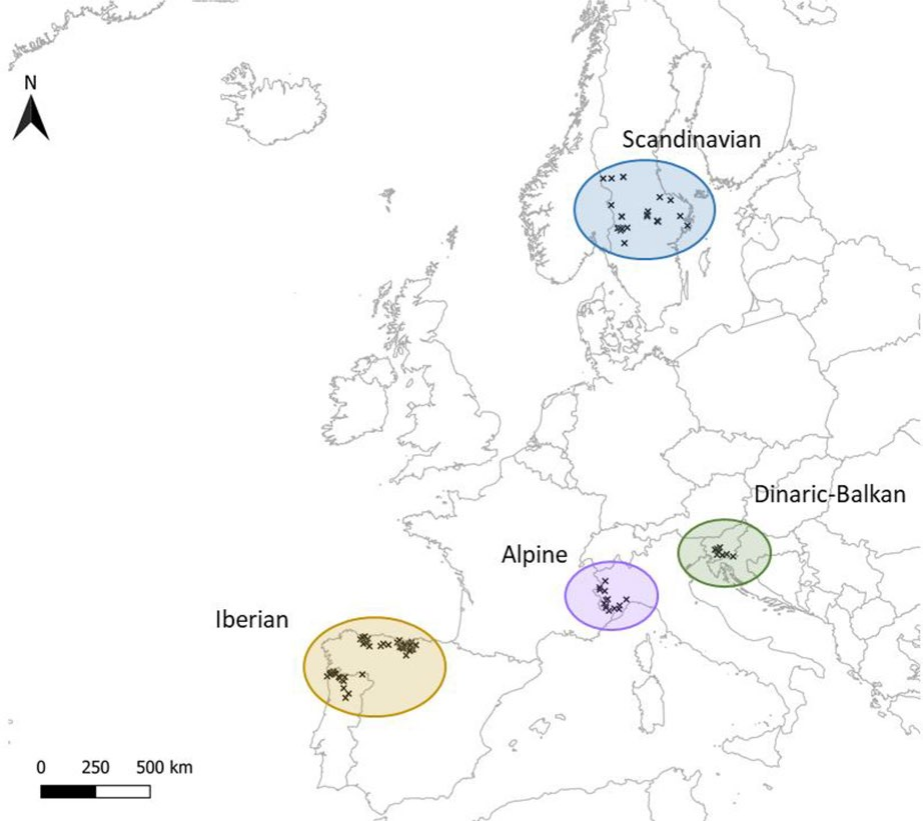
Geographical distribution of the death or live-trapping sites of the grey wolf hair samples analyzed. European wolf populations enclosed by circles.

Hair samples (n = 259) were obtained from 133 wolves captured for scientific purposes, legally harvested or found dead in Spain (n = 146, from 77 wolves), Portugal (n = 34, 14 wolves), Sweden (n = 38, 19 wolves), Italy (n = 26, 13 wolves), and Slovenia (n = 15, 10 wolves) (Fig. 1). The samples were collected from September 2014 to January 2020.

**Figure 2:**
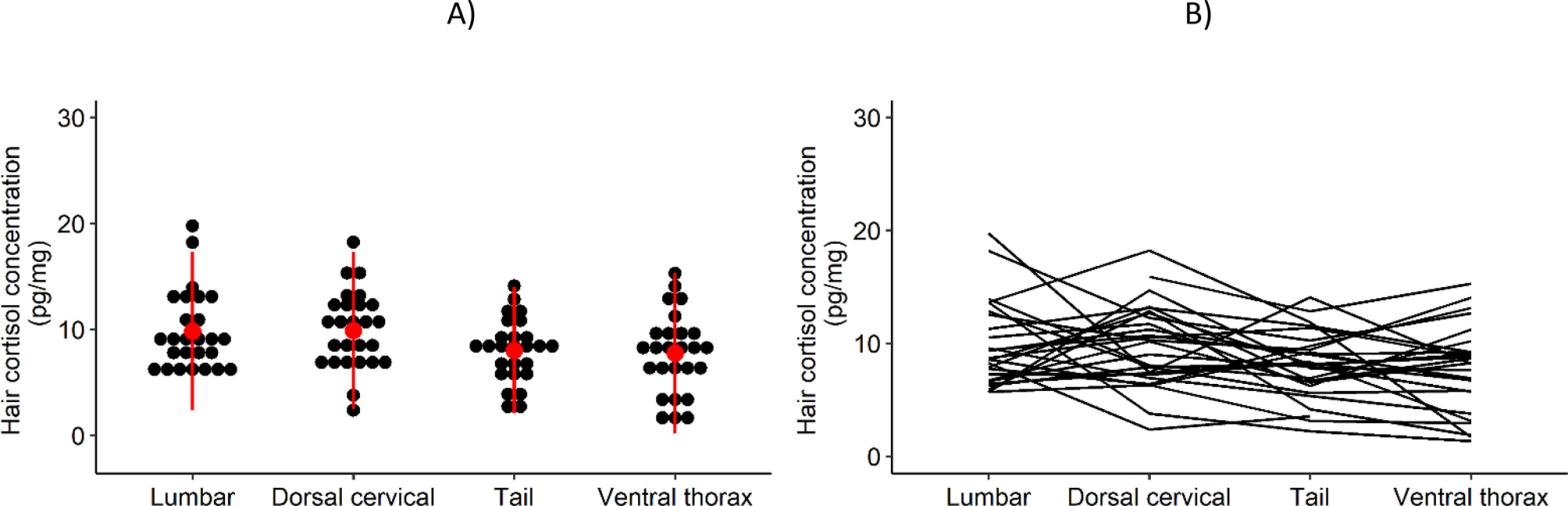
Hair cortisol concentration by body region. (**A**) Mean ± 2 standard deviations (in red) of hair cortisol in paired samples from 27 wolves with 4 body regions analyzed per individual. (**B**) Variation of HCC between body regions of individual wolves.

The tail and ventral thorax regions, but not dorsal thorax, showed significantly lower HCC than the lumbar region (Table 1 and Fig. 2A,B), after the exclusion of 4 outliers. All subsequent analyses were performed for lumbar hair samples only.

**Table 1:** Summary of the linear mixed model of hair cortisol concentration by body region. Individual wolf included as random effect, lumbar body region as reference class. Results from 27 wolves with 4 body regions each (4 outliers excluded).

Variable	β	Standard error (β)	95% Confidence interval (β)		df
			Low	High	
**Fixed effects**					
Intercept	9.952	0.702	8.612	12.270	75.7
**Body region**					
Dorsal cervical	− 0.119	0.811	− 1.709	1.471	73.1
Tail	− 1.967	0.811	− 3.557	− 0.377	73.1
Ventral thorax	− 2.324	0.811	− 3.914	− 0.734	73.1
**Random effect**					
Variance	4.412				
Standard deviation	2.101				
N wolf	27				
N samples	104

The mean HCC of lumbar guard hair, excluding 2 outliers (see “Methods”), was 11.64 ± 9.65 pg/mg, ranging 1.58–108.81 pg/mg. The most supported model (Model 1, Table S4), with a weight 0.122, included the fixed effects sex, age, population, body size (total length), and cause of death/capture (Table 2). The cumulative weight of the 4 models with ΔAICc < 2 was 0.320. The sum of the variable weights in the 4 models with ΔAICc < 2 (Table S4) was 1 for age, population, body size, and cause death, 0.85 for sex, 0.32 for evaporation protocol, and 0.15 for hair length. Model 1 conditional R^2^ = 0.232, and the null model’s ΔAICc = 24.5, supporting the ability of the former to explain the variation in HCC.

**Table 2:** Summary of the most supported linear mixed model of the determinants of hair cortisol concentration. Results from Model 1 in Table S4.

Variable	β	Standard error (β)	95% Confidence interval (β)		df
			Low	High	
**Fixed effects**					
Intercept	10.688	0.856	9.010	12.366	50.681
**Sex**					
Males	0.722	0.876	− 0.995	2.439	99.204
Unknown	2.576	4.470	− 6.185	11.337	99.123
**Age**					
Subadults	− 1.453	0.973	− 3.360	0.454	93.085
Juveniles	− 0.273	1.796	− 3.793	3.247	97.429
Unknown	− 0.935	2.545	− 5.923	4.053	99.023
**Population**					
Alpine	1.721	1.768	− 1.744	5.186	12.389
Dinaric-Balkan	3.643	1.808	0.099	7.187	92.999
Scandinavian	3.167	1.261	0.695	5.639	90.521
**Cause of death**					
Subacute	0.529	1.632	− 2.670	3.728	99.507
Chronic	− 5.043	4.640	− 14.137	4.051	99.761
Live trapping	− 2.852	4.409	− 11.494	5.790	98.541
Unknown	2.409	1.492	− 0.515	5.333	99.913
Total length	− 0.141	0.043	− 0.225	− 0.057	99.942
**Random effect**					
Variance	1.039				
Standard deviation	1.019				
N samples	114				
N month	12				

**Figure 3:**
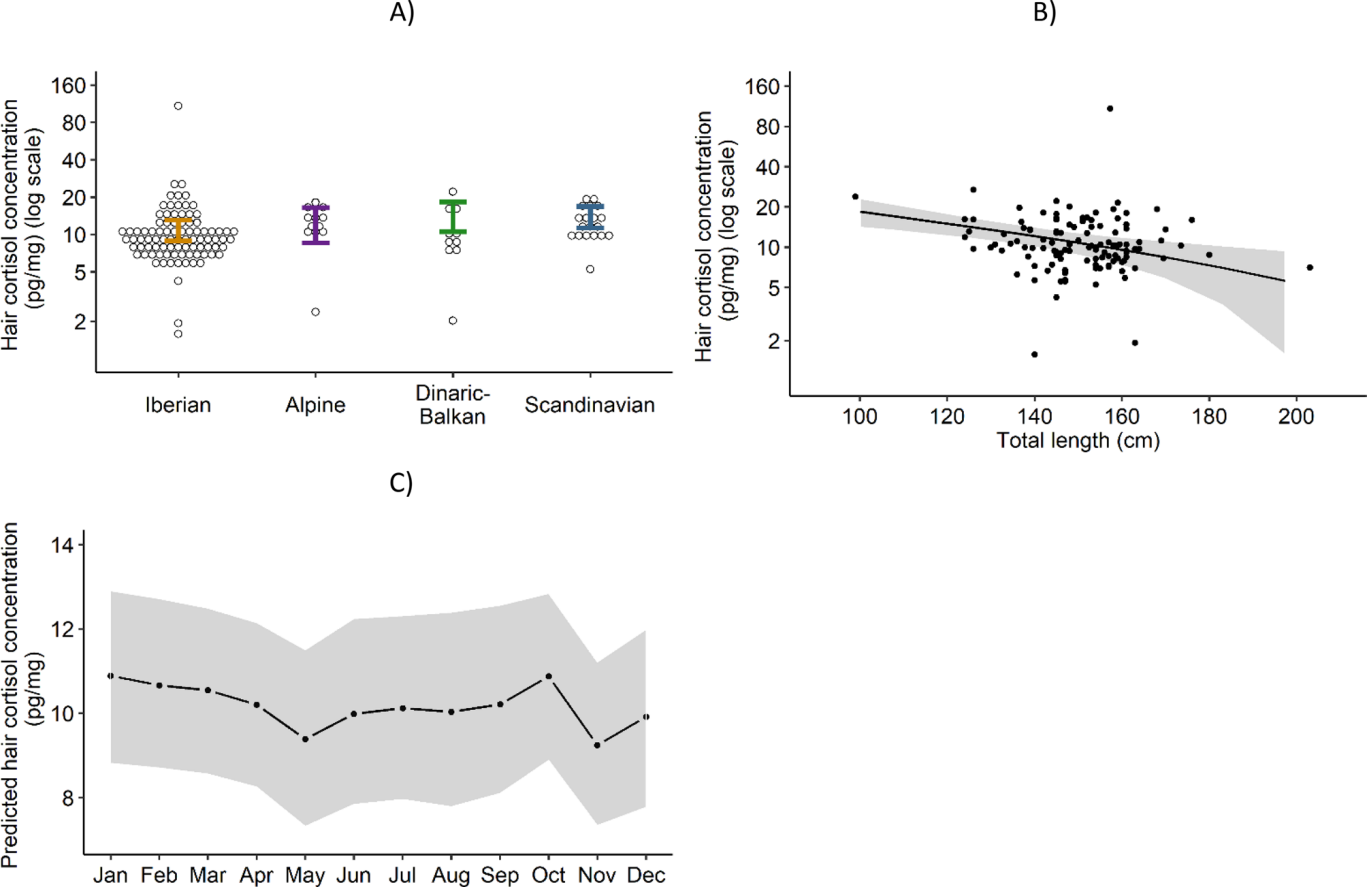
Hair cortisol concentration observed and predicted by the linear mixed model. Hair cortisol concentration by: (**A**) wolf population: observed (dots) and predicted by the linear mixed model (95% confidence interval as colored error bars). (**B**) Total length: observed (dots) and predicted by the linear mixed model with 95% confidence interval as shaded grey area. (**C**) Month: 95% confidence interval as shaded grey area. Predictions of the most supported model for wolves of the reference classes, 95% confidence intervals include fixed and random effects.

The most supported model revealed significantly higher HCC in the Dinaric-Balkans and Scandinavian populations (Fig. 3A). Hair cortisol concentration showed a significant negative relationship with total length (Fig. 3B), and higher values in the winter and lowest in May and November (Fig. 3C).

**Figure 4:**
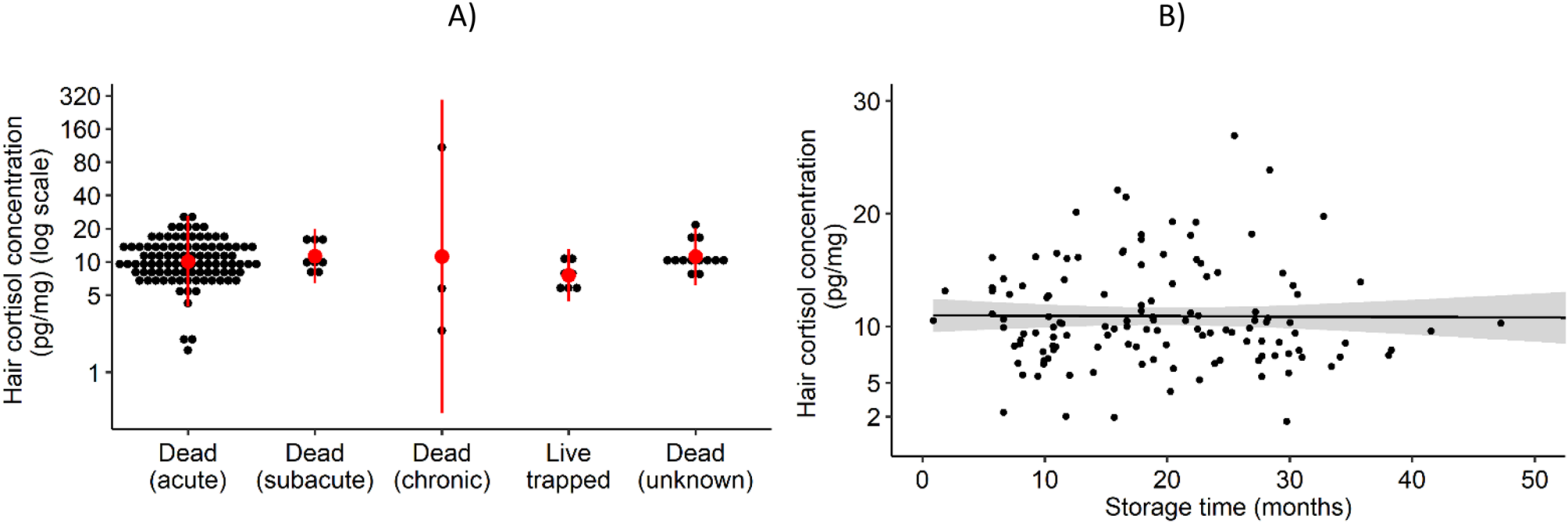
Hair cortisol concentration by selected methodological determinants. (**A**) Cause of death/capture: mean ± 2 standard deviations (in red) of hair cortisol concentration measured in lumbar guard hair samples. (**B**) Sample storage: linear regression between measured hair cortisol concentration and time from death/capture to cortisol extraction with the 95% confidence interval as shaded grey area.

No single cause of death/capture showed a significant relationship with HCC, but samples assigned the ‘chronic death’ category tended for extreme high and low values (Fig. 4A). No evidence was found for lower HCC in samples stored for up to 4 years (Fig. 4B).

## Discussion

The mean HCC in lumbar guard hairs of wolves from the four European populations studied (11.6 ± 9.7 pg/mg) was lower than that reported for wolves from the Canadian tundra–taiga (16.5 pg/mg) and northern boreal forest (13.5 pg/mg)^15^. It was suggested that the degree of legal lethal removal by humans (licensed hunting or population control) was reflected on the HCC of Canadian wolf populations, possibly mediated by the increased social instability of wolf populations subjected to intense harvest^15^. Therefore, the mean HCC in our sample, being lower than in the less heavily harvested Canadian boreal forest population, could imply a lower intensity of legal lethal removal of the European wolf populations studied. Bryan et al.^15^ do not quantify the proportion of their study populations that is harvested but mention it can be up to 50% annually. In the same Canadian province of Alberta, 34% of the wolf population was estimated to be legally harvested every year^21^. In Europe, the disappearance rate of Scandinavian wolves was estimated at 23%, with legal harvest amounting to 5–15% annually^22^. In the Alpine wolf population, annual mortality was estimated at 10–25%^23,24^. The apparently lower human-related harvest of wolves in Europe, compared to Canada, could explain the lower HCC in our study.

Wolves were fully protected in Portugal and Italy but subjected to varying degrees of legal harvest in Spain, Slovenia, Sweden as well as in other countries of the Alpine, Dinaric-Balkan, and Scandinavian population ranges that were not sampled in this study^19^. The populations showing significantly higher predicted HCC (Dinaric-Balkan and Scandinavian) were subjected to legal harvest, while the partially protected Iberian and Alpine populations did not significantly differ between them. Even fully protected wolf populations in Europe experience high levels of poaching^22,25,26^, which could dampen the differences in the legal wolf removal and in HCC across the four European populations studied. Furthermore, extrinsic factors not addressed in this study, such as wolf density, prey availability and non-lethal human disturbance can also influence the HCC^11,13^. The potential of HCC as biomarker of the intensity of legal and illegal human-related harvest of wolves across Europe needs to be further established.

On the other hand, the differences in HCC between European and Canadian wolves could also be caused by slightly different methodologies employed by Bryan et al*.*^15^, such as a non-standardized body region for the collection of hair samples, hair grinding by ball mill or a different commercial ELISA. The grinding method was shown to have a significant effect on the amount of cortisol extracted from hair, possibly mediated by matrix and surface area mechanisms^27,28^. In our study, it was decided to use a closed-tube method, with the advantage of preventing loss of hair powder and sample contamination that can occur when using a ball mill^27^. Moreover, large differences in HCC according to the ELISA protocol utilized have also been described^29,30^ and could contribute to explain the differences in measured HCC to Canadian wolves^15^. Despite the unplanned change in the evaporation protocol (nitrogen gas stream vs suction hood evaporation) accounting for a non-significant effect on HCC, this was the most relevant methodological variable (weight 0.32). Full standardization of the analytical protocol is strongly recommended.

Our results show difference in HCC across the body regions from which hair was collected. Results from other studies are inconsistent, as differences in HCC according to body region have been reported in some species but not in others^11^. The main mechanism hypothesized for the incorporation of glucocorticoids into growing hair is via the blood vessels and eccrine and sebaceous glands that surround the hair follicle^4^. Guard hair from different body regions may present disparate periods and rates of growth and therefore cortisol will not be incorporated equally throughout the body^13^. Wolves seem to have one annual molt, beginning in late spring when the old coat is shed, and the new summer coat grows continuously through fall and winter^18^. Relating hair samples to a specific period is more challenging in species with poorly known molt patterns^15^. Our results support that hair should be collected from standardized body regions to improve inference from HCC.

The small number of wolves in this study showing viral, bacterial, or ectoparasitic infections or neoplasia (n = 5, including 1 outlier excluded from Model 1—see “Methods”), some of which caused their deaths, tended to present extreme HCC compared to apparently healthy wolves. Chronic diseases have been shown to cause major increases in HCC in many species^11^ and higher levels were found in chronic compared to acute diseases in cattle^31^. The highest HCC in our sample were associated with sarcoptic mange, which can course for many weeks to months in individual wolves^32^. In contrast, death by events likely lasting minutes to a few days or live trapping did not translate into significant alterations to HCC. Together, these results support the hypothesis that HCC reflects cortisol levels in the organism in a retrospective time frame of several weeks^4,11^.

No evidence was found for lower HCC in samples stored for up to 4 years, suggesting that archived specimens could be used to provide insights into the long-term dynamics of chronic stress in wolf populations. Nevertheless, this study was not designed to evaluate the effect of storage time on HCC, which would be better accomplished in a longitudinal design. Other studies report inconsistent results on this potentially confounding variable, from a significant negative effect of storage time in the Egyptian mongoose *Herpestes ichneumon*^33^ to higher HCC in polar bear *Ursus maritimus* hair stored for > 100 years than in recent samples^14^.

Males showed non-significantly higher HCC than females. Other studies consistently report non-significant differences between sexes, with higher HCC in females^15,35^ or males^34^. Younger age classes, particularly subadults, showed a tendency for lower HCC than the adults. The same age pattern was reported for HCC and fecal cortisol metabolites in North American and European wolves^34,35^.

The monthly variation in HCC predicted by the model is consistent with the annual life cycle of wolves and with the results of fecal glucocorticoid metabolites^34–36^. The highest HCC was found in the winter, at the start of the mating season^37^ and could be a consequence of increased social instability related to sexual and territorial behavior^11,34^. Wolves are strict seasonal monoestrous breeders^18^. It has been shown in other species that cortisol can play a role in reproduction by inhibiting the action of oestradiol and luteinizing hormone and, consequently, inhibiting female receptivity to mating and ovulation^38^. The surge in cortisol during the mating season could thus be a contributing mechanism to regulate reproduction within wolf packs, where usually only one pair breed, even when other potential breeders are present^18^. The lowest HCC was found in May, coinciding with parturition, during which intraspecific competition and social instability are lessened^37^. Between May and October the HCC progressively increases which could be related to the growing effort to supply pups. The peak in HCC in October coincides with juveniles leaving their homesites and start travelling with the pack^36,39^.

Our results support the suitability of hair cortisol concentration as biomarker of chronic stress in wolves, suggesting that it allows the assessment of cortisol levels in a retrospective time frame of weeks to months, as postulated for this biological matrix^4,6^. This is supported by the observation that causes of death with an expected duration of days to weeks do not translate in higher HCC, while the small number of wolves with chronic infections, particularly sarcoptic mange, showed the highest values. The reliability of this approach is further supported as the observed annual cycle of HCC is consistent with the levels of chronic stress inferred from the wolves’ life cycle and other studies measuring fecal cortisol metabolites^34–36^. The sex and age patterns are also similar to those reported in other studies with wolves, measuring hair cortisol or fecal cortisol metabolites^15,34–36^. This study supports that sampling (e.g. body region, cause of death) and analytical (e.g. methanol evaporation protocol and length of hair) techniques should be standardized or accounted for in the statistical analysis to improve inference from HCC data.

## Methods

### Collection of wolf hair samples

Hair samples were collected by researchers from opportunistically found-dead wolves upon standard necropsy (all the Alpine and part of the Iberian samples) or in the field (all the Dinaric-Balkan and most of the Iberian samples), or from legally harvested wolves (only in the Scandinavian population). At the time of sample collection, wolves were legally harvested in Sweden, Slovenia, and Spain, and under total protection in Portugal and Italy. Hair samples were collected from four body regions, when possible: lumbar (n = 133), dorsal cervical (n = 66), tail (n = 33) and ventral thorax (n = 27) (Tables S1 and S2). The hair was cut as close as possible to the skin with scissors to avoid collecting hair follicles, but in some samples, hairs were pulled from the carcass. Samples were stored at room temperature in paper envelopes. Age, sex, date, and cause of death/capture, geographical location, body mass, and total length were obtained for most of the wolves.

Age was estimated by the dental eruption and wear or cementum age analysis and classified as ‘juveniles’ (< 1 year old), ‘subadults’ (1–2 years), ‘adults’ (> 2 years)^40^, or ‘unknown’. Sex was assessed by inspection of genitalia. Causes of death were classified as ‘acute’, likely lasting minutes to hours (vehicle accident and legal or illegal shooting); ‘subacute’, likely lasting hours to days (drowning, poisoning, trapping and intraspecific aggression); ‘chronic’, likely lasting several weeks (infectious diseases—canine distemper, canine parvovirosis, leptospirosis; sarcoptic mange; or neoplastic diseases) or ‘unknown’. Total length was obtained by measuring with metric tape (1 mm precision) the distance from snout to the distal end of the last tail vertebrae. The body mass was measured with 100 g precision with scales.

The detailed protocol for the handling of wolves live trapped in the scope of ecological and conservation studies (n = 7, all from the Iberian population) has been previously described^5^. Traps were monitored twice every day, in the early morning and late afternoon, hence the duration of restraint after capture was unknown for 8 wolves, potentially up to 12 h. Trap-alarms were deployed in the capture of 2 wolves, with 41 and 70 min intervals between activation of the alarm and administration of the drugs. Live trapping was conducted under permits issued by the nature conservation authorities of Portugal (*Instituto de Conservação da Natureza e das Florestas*: 338/2007/CAPT, 258/2008/CAPT, 286/2008/CAPT, 260/2009/CAPT, 332/2010/MANU, 333/2010/CAPT, 336/2010/MANU, 26/2012/MANU, and 72/2014/CAPT) and Spain (*Dirección Xeral de Conservación da Naturaleza, Xunta de Galicia*: E-0020/13-PNPE, 095/2013; *Consejería de Medio Ambiente, Principado de Asturias*: 31/08/2017-BOPA 05/09/17) and according to European Union directives on the protection of animals used for scientific purposes (Directive 2010/63/EU) and international wildlife standards^41,42^. The study was undertaken in compliance with the ARRIVE guidelines^43^.

### Cortisol extraction

The protocol for the extraction of cortisol from the hair was adapted from previously described procedures^15,27^. Forty mg of guard hairs were separated from the undercoat and placed in 15 ml falcon tubes. Hair follicles were cut whenever found in the sample. For each sample, the length of three intact hairs was recorded. The samples were washed twice with 40 µl of distilled water/mg hair and three times with the same amount of isopropanol. In each washing step, the samples and washing solution were vortexed, the supernatant discarded, and the hair dried using clean paper towels. After the final wash, samples were dried overnight at room temperature and 30 mg of hair cut into a 2 ml polypropylene screw cap plastic tube with five 4 mm steel beads added to each tube.

The hair was ground to a fine powder in a FastPrep sample homogenizer (MP Biomedicals, USA) for four times 1 min at 6.0 m/s. 50 µl methanol/mg hair were added to each sample and sonicated for 30 min at 50 Hz at 50 °C. The samples were incubated for 18 h at 50 °C in an orbital shaker at 160 rpm, centrifuged for 15 min at 14,000*g* at 20 °C, and 1000 µl of supernatant was collected to a screw cap glass chromatography vial and dried at room temperature in a gentle stream of nitrogen gas. Due to restrictions on laboratory use during the SARS-Cov-2 pandemic, some batches of samples were instead evaporated overnight on a suction hood. This unexpected change in the methanol evaporation protocol was recorded and accounted for in the statistical analysis.

### Cortisol quantification

A commercial competitive ELISA kit (Cortisol free in Saliva ELISA, Demeditec, Germany) was used to quantify the concentration of cortisol, following the manufacturer’s instructions. The kit plate wells are provided coated with polyclonal rabbit antibody against cortisol, and cortisol-horseradish peroxidase was used as conjugate. According to the manufacturer, the cross-reactivity of the test to selected steroids is low (Table S3), the intra-assay variation is 3.8–5.8% and the inter-assay variation is 6.2–6.4%. Samples, standards, and controls were tested in duplicate.

The 4-parameter standard curve was calculated from the log-transformed cortisol concentration of the standard solutions and their measured OD_450_^44^. Standard curves were estimated using the software GraphPad Prism 6.04 (GraphPad Software, La Jolla, California USA), and yielded an average R^2^_adjusted_ = 0.991 (range 0.968–0.999). The cortisol concentration of the reconstituted samples was estimated from the standard curve and converted to cortisol concentration as picograms (pg) of cortisol/mg of guard hair.

Intra and inter-assay coefficients of variation were estimated for six ELISA assays of 37–40 samples each. The low and high controls included in the kit were used to estimate the inter-assay coefficient of variation and the duplicate runs of each sample were used to estimate the intra-assay coefficient of variation. Linearity was assessed by two-fold dilutions (1:1, 1:2, 1:4 and 1:8) of 4 extracted samples, comparing the expected and observed concentrations. Recovery was assessed by spiking 6 ground hair samples with known concentrations of cortisol (50, 25, 12.5, 6.25 pg/mg, and no spiking), comparing the expected and observed concentrations.

The intra-assay coefficient of variation of the ELISA assays ranged from 6.50 to 9.97% (average 7.66%). The inter-assay coefficient of variation was 11.54% for the low concentration controls and 9.08% for the high concentration controls (average 10.31%). Assay linearity was 91% for the 1:2 dilution, 103% for 1:4, and 117% for 1:8 (average 103%). The recovery of cortisol averaged 94%, being 73% for the 50 pg/mg spiked samples, 74% for 25 pg/mg, 95% for 12.5 pg/mg, and 113% for 6.25 pg/mg.

### Determinants of hair cortisol concentration

The potential determinants of HCC investigated included wolf intrinsic variables: sex, age, body condition, body structural size, month of death/capture, and wolf population. The scaled mass index was selected as a measure of body condition^45^ and estimated from the log-transformed body weight (g) and total length (mm). Log-transformed total length was used as an indicator of body structural size^46^. Samples were assigned to the Iberian, Alpine, Dinaric-Balkan, or Scandinavian wolf populations^16^ from the geographical location of the death or live-trapping sites (Fig. 1).

The relationship between HCC and additional variables related to the sampling procedure or to the work conducted in the laboratory (length of hair used for cortisol extraction, sample storage time, body region, cause of death/capture, and methanol evaporation protocol), herein referred to as methodological variables, was also investigated as potential confounding variables. Sample storage time was the period in months between death/capture and cortisol extraction. In those samples for which only the year of death was available, 30 June was assigned as the date of death, solely to estimate storage time. All continuous variables were standardized to their z-scores.

### Statistical analysis

First, the effect of body region was investigated by a linear mixed model with HCC as the dependent variable, and the independent variables body region, as a categorical fixed effect, and individual wolf, as a random effect. The lumbar region was set as the reference class as it was the most represented in our sample (Table S1). Data from 27 wolves for which samples were available from all 4 body regions were used in this analysis. Four outliers in the dataset violated the assumption of normality in the residuals of the model comparing HCC across body regions (Fig. S1A) and were excluded from this model’s dataset (Fig. S1B).

Second, the effect of intrinsic and methodological variables on HCC from the lumbar body region was investigated by another linear mixed model with sex, age, body condition, body structural size (standardized log-transformed total length), cause of death/capture, wolf population, hair length, sample storage time, and methanol evaporation protocol as fixed effect independent variables. The month of death/capture was included as a random effect. Reference classes for the categorical variables were set as female, adult, acute death, Iberian population, and methanol evaporation by nitrogen gas stream. Two outliers in the dataset violated the assumption of normality in the residuals of the model (Full model, Table S4) and were excluded from this analysis (Fig. S1C,D).

The goal of this analysis was to assess the relationship between HCC and wolf intrinsic variables, controlling for the potential confounding effect of the methodological variables. Starting from the full model (Table S4), models including all possible combinations of variables were ranked by their AICc using the package “MuMIn”^47^ in R 3.6.1^48^. The most supported model was selected for inference and models with ΔAICc < 2 are reported in Table S4.

The HCC predicted by the most supported model was estimated using the package “merTools”^49^. The function “predictInterval” was used, which fits multivariate normal distributions to the random and fixed effects. 1000 values were sampled from these distributions for each category of the random (month) and fixed effects, capturing the full uncertainty in predictions as 95% confidence intervals.

Linear mixed models were fitted using the package “lme4”^50^ in R 3.6.1^48^. The correlation between fixed effects was estimated with a threshold for acceptance of 0.700. The conditional R^2^ of the model was estimated according to Nakagawa and Schielzeth^51^ implemented in the package “MuMIn”^47^. The assumption of normality of the model residuals was checked by inspection of quantile–quantile plots. Graphics were produced using the R package “ggplot2”^52^.

## Supplementary Information

Supplementary Information.

## Data Availability

The datasets generated during the current study are available from the corresponding authors on reasonable request.
