# Supplementary material for: Hair cortisol concentration reflects the life cycle and management of grey wolves across four European populations
Source: Sci Rep. 2022 Apr 5;12:5697. doi: 10.1038/s41598-022-09711-x (PMC8982655; doi:10.1038/s41598-022-09711-x)

**Supporting Information**

**Hair cortisol concentration reflects the life cycle and management of grey wolves across four European populations**

Patrícia Pereira, Núria Fandos Esteruelas, Mónia Nakamura, Helena Rio-Maior, Miha Krofel, Alessia Di Blasio, Simona Zoppi, Serena Robetto, Luis Llaneza, Emilio García, Álvaro Oleaga, José Vicente López-Bao, Manena Fayos Martinez, Jasmine Stavenow, Erik Agren, Francisco Álvares, Nuno Santos

Table S1 – Summary characterization of the sample by sex, age, body region and cause of death/capture.

Table S2 – Summary characterization of the sample by month of death/capture.

Table S3 – Cross-reactivity of the ELISA test to selected steroids, as reported by the manufacturer.

Table S4 – Summary of the linear mixed models with backwards stepwise elimination of methodological independent variables.

Fig. S1 - Validation plots of the most supported models.

**Table S1 – Summary characterization of the sample by sex, age, body region and cause of death/capture.**

| **Wolf population** | **Age (n wolves)** | | | | **Sex (n wolves)** | | | **Body region (n samples)** | | | | **Capture/causes of death (n wolves)** | | | | |
| --- | --- | --- | --- | --- | --- | --- | --- | --- | --- | --- | --- | --- | --- | --- | --- | --- |
|  | Juvenile | Subadult | Adult | Unknown | Female | Male | Unknown | Lumbar | Dorsal cervical | Tail | Ventral | Live trapping^1^ | Dead | | | |
|  |  |  |  |  |  |  |  |  |  |  |  |  | Acute^2^ | Subacute^3^ | Chronic^4^ | Unknown |
| Iberian | 7 | 31 | 48 | 5 | 42 | 48 | 1 | 91 | 29 | 33 | 27 | 7 | 63 | 5 | 4 | 12 |
| Alpine | 5 | 4 | 4 | 0 | 7 | 6 | 0 | 13 | 13 | 0 | 0 | 0 | 10 | 2 | 1 | 0 |
| Dinaric-Balkan | 2 | 4 | 3 | 1 | 3 | 5 | 2 | 10 | 5 | 0 | 0 | 0 | 9 | 0 | 0 | 1 |
| Scandinavian | 0 | 3 | 16 | 0 | 7 | 11 | 1 | 19 | 19 | 0 | 0 | 0 | 17 | 2 | 0 | 0 |
| Total | 14 | 42 | 71 | 6 | 59 | 70 | 4 | 133 | 66 | 33 | 27 | 7 | 99 | 9 | 5 | 13 |

^1^ Live trapping for scientific purposes

^2^ Vehicle accident or legal or illegal shooting

^3^ Drowning, poisoning, trapping or intraspecific aggression

^4^ Diseases: infectious (canine distemper, canine parvovirosis, leptospirosis), parasitic (sarcoptic mange), or neoplastic

**Table S2 – Summary characterization of the sample by month of death/capture.**

| **Wolf population** | **Month (n wolves)** | | | | | | | | | | | | |  |
| --- | --- | --- | --- | --- | --- | --- | --- | --- | --- | --- | --- | --- | --- | --- |
|  | January | February | March | April | May | June | July | August | September | October | November | December | Unknown | |
| Iberian | 5 | 7 | 11 | 11 | 7 | 5 | 3 | 5 | 6 | 13 | 9 | 7 | 2 | |
| Alpine |  |  |  |  |  |  |  |  |  |  |  |  | 13 | |
| Dinaric-Balkan |  |  |  | 1 | 1 | 1 |  |  | 1 | 1 | 4 |  | 1 | |
| Scandinavian | 7 |  | 2 | 3 | 2 |  |  |  |  |  | 2 | 3 |  | |
| Total | 12 | 7 | 13 | 15 | 10 | 6 | 3 | 5 | 7 | 14 | 15 | 10 | 16 | |

**Table S3 – Cross-reactivity of the ELISA test to selected steroids, as reported by the manufacturer.** ELISA kit ‘Cortisol free in Saliva ELISA’, Demeditec, Germany.

| **Steroids** | **Cross-reactivity (%)** |
| --- | --- |
| Testosterone | < 0.1 |
| Corticosterone | 5.2 |
| Cortisone | 0.2 |
| 11-Deoxycorticosterone | 0.4 |
| 11-Deoxycortisol | 10.4 |
| Dexamethasone | < 0.1 |
| Estriol | < 0.1 |
| Estrone | < 0.1 |
| Prednisolone | 63.4 |
| Prednisone | < 0.1 |
| Progesterone | < 0.1 |
| Danazole | < 0.1 |
| Pregnenolone | < 0.1 |
| Estradiol | < 0.1 |

**Table S4 – Summary of the linear mixed models selected by their AICc.** Models with ΔAICc<2 from the most supported model, full and null models included.

| **Model** | **Variables included in the model** | **AICc** | **ΔAICc** | **Model weight** |
| --- | --- | --- | --- | --- |
| 1 | HCC ~ sex + age + population + body size + (1\|month) + cause death | 646.22 | 0 | 0.122 |
| 2 | HCC ~ sex + age + population + body size + (1\|month) + cause death + evaporation protocol | 646.52 | 0.34 | 0.103 |
| 3 | HCC ~ age + population + body size + (1\|month) + cause death | 648.10 | 1.88 | 0.048 |
| 4 | HCC ~ sex + age + population + body size + (1\|month) + cause death + hair length | 648.12 | 1.89 | 0.047 |
| Full | HCC ~ sex + age + population + body size + SMI + (1\|month) + cause death + hair length + evaporation protocol + storage time | 655.67 | 9.45 | 0.001 |
| Null | HCC ~ (1\|month) | 670.76 | 24.54 | 5.7 x 10^-7^ |

HCC: hair cortisol concentration

SMI: scaled mass index

AICc: Akaike's Information Criterion corrected

**Figure S1 – Validation plots of the most supported models.** Normality of the residuals of the linear mixed model of HCC by body regions for the whole dataset A) and excluding 4 outliers B). Normality of the residuals of the full linear mixed model for the whole dataset C) and excluding 2 outliers D). Relationship between the predicted hair cortisol concentration observed and predicted by Model 1 E).

A)


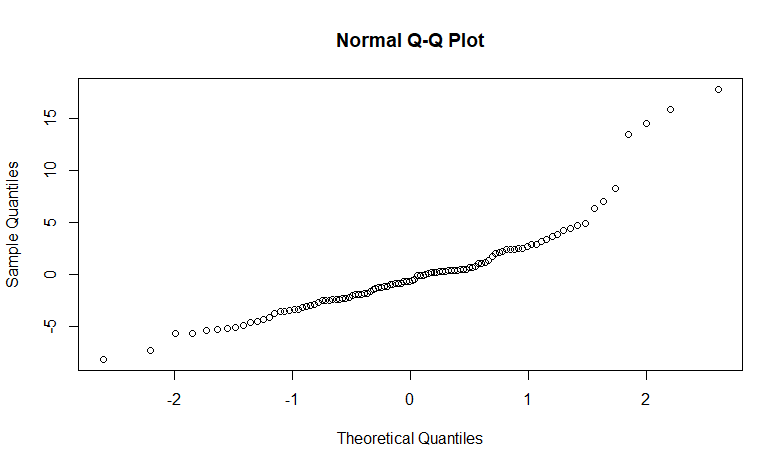


B)


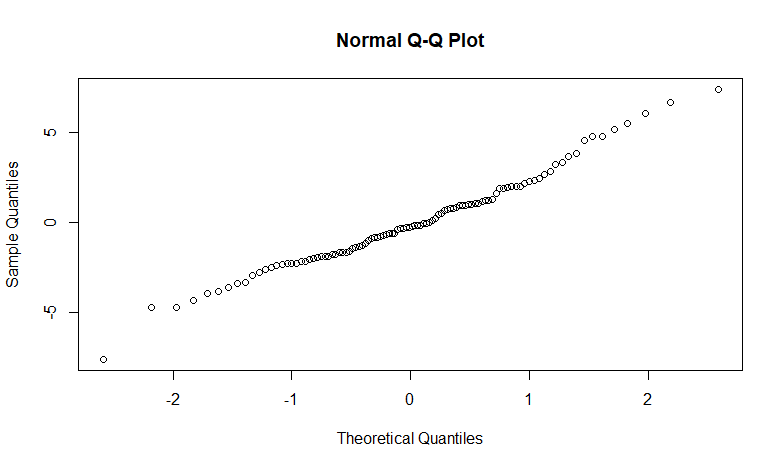


C)


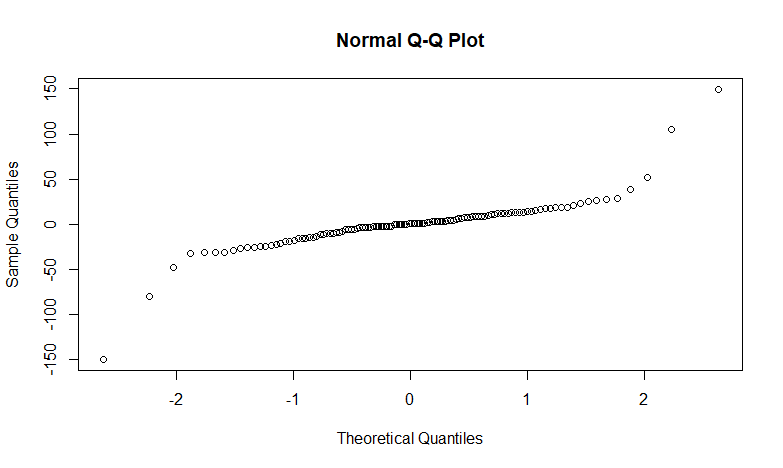


D)


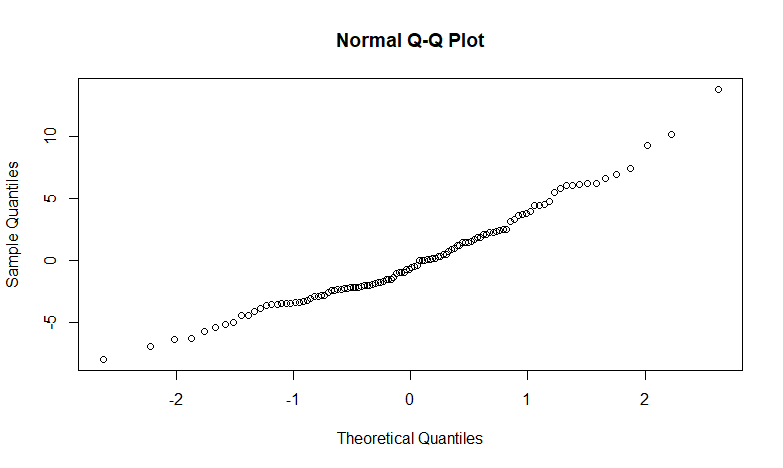


E)


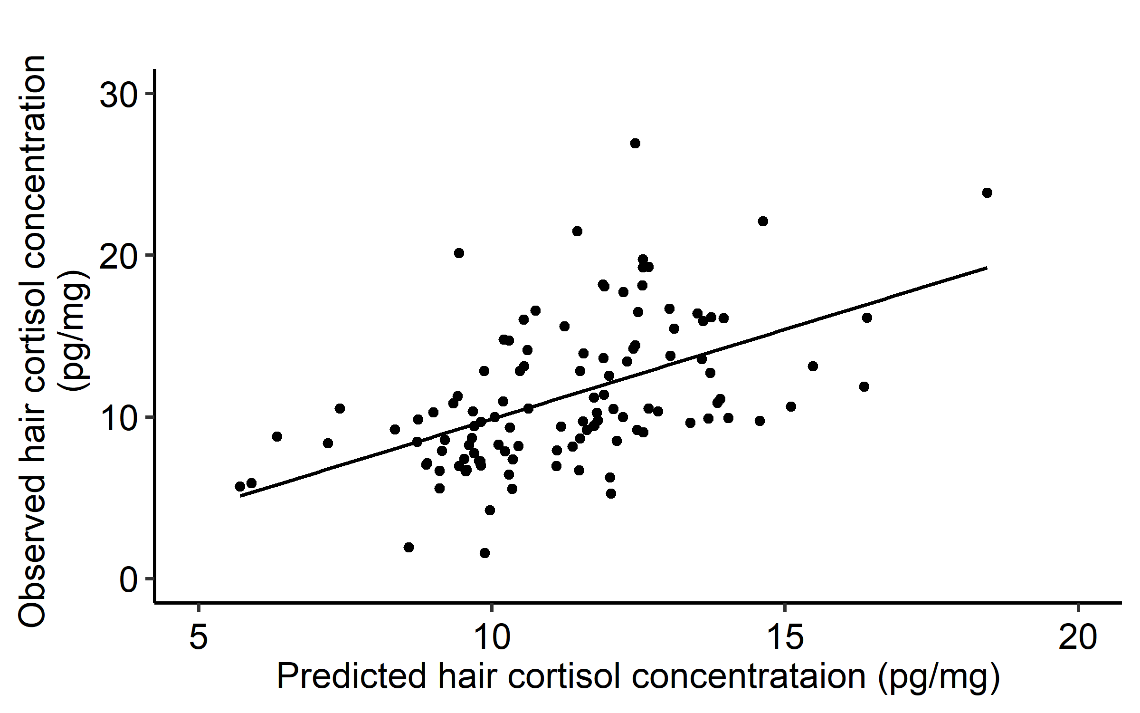

Supplement: Supplementary file 1 — Supplementary Information. [file 41598_2022_9711_MOESM1_ESM.docx]
